# Therapeutic Influence on Important Targets Associated with Chronic Inflammation and Oxidative Stress in Cancer Treatment

**DOI:** 10.3390/cancers13236062

**Published:** 2021-12-01

**Authors:** Margarita Neganova, Junqi Liu, Yulia Aleksandrova, Sergey Klochkov, Ruitai Fan

**Affiliations:** 1 Department of Radiation Oncology, The First Affiliated Hospital of Zhengzhou University, Erqi, Zhengzhou 450000, China; neganovam@ipac.ac.ru (M.N.); fccliujq@zzu.edu.cn (J.L.); 2 Institute of Physiologically Active Compounds, Russian Academy of Sciences, 142432 Chernogolovka, Russia; aleksandrova@ipac.ac.ru; 3 Cancer Center, The First Affiliated Hospital of Zhengzhou University, Zhengzhou 450052, China

**Keywords:** cancer, inflammation, oxidative stress, metabolism, cytokines, HIF1α, TNFα, Nrf2

## Abstract

**Simple Summary:**

There is no doubt that the need for new effective methods of cancer treatment remains challenging, as cancer is the second cause of death based on the number of cases in the world. In this review, we investigated the role of one of the leading determinants in the development and progression of various types of cancer—oxidative stress and inflammation, as well as clinical and experimental data from the studies of promising drugs of natural origin, such as flavonoids, that target these stages of oncogenesis. This can all help in the expansion and systematization of the existing knowledge regarding the fight against cancer, the facilitation of the development of effective anti-cancer drugs, and the progression of research in this field, in order to improve the treatment of these disorders.

**Abstract:**

Chronic inflammation and oxidative stress are the interconnected pathological processes, which lead to cancer initiation and progression. The growing level of oxidative and inflammatory damage was shown to increase cancer severity and contribute to tumor spread. The overproduction of reactive oxygen species (ROS), which is associated with the reduced capacity of the endogenous cell defense mechanisms and/or metabolic imbalance, is the main contributor to oxidative stress. An abnormal level of ROS was defined as a predisposing factor for the cell transformation that could trigger pro-oncogenic signaling pathways, induce changes in gene expression, and facilitate accumulation of mutations, DNA damage, and genomic instability. Additionally, the activation of transcription factors caused by a prolonged oxidative stress, including NF-κB, p53, HIF1α, etc., leads to the expression of several genes responsible for inflammation. The resulting hyperactivation of inflammatory mediators, including TNFα, TGF-β, interleukins, and prostaglandins can contribute to the development of neoplasia. Pro-inflammatory cytokines were shown to trigger adaptive reactions and the acquisition of resistance by tumor cells to apoptosis, while promoting proliferation, invasion, and angiogenesis. Moreover, the chronic inflammatory response leads to the excessive production of free radicals, which further aggravate the initiated reactions. This review summarizes the recent data and progress in the discovery of mechanisms that associate oxidative stress and chronic inflammation with cancer onset and metastasis. In addition, the review provides insights for the development of therapeutic approaches and the discovery of natural substances that will be able to simultaneously inhibit several key oncological and inflammation-related targets.

## 1. Introduction

The treatment of cancer has originated from the 17th century [1] and includes a great amount of chemotherapy [2,3,4], radiotherapy [5,6,7], and surgical [8] methods. However, despite the large number of existing approaches for the treatment of malignant neoplasms, their effectiveness in some cases is limited due to the development of multidrug resistance [9,10], the occurrence of recurrent medical conditions [11], and the indiscriminate death of tumor cells affecting the healthy body microenvironment [12,13,14]. According to the estimates of the World Health Organization, cancer is the second cause of death in the world, second only to cardiovascular diseases [15]. According to the International Agency for Research on Cancer, in 2020, 19.3 million new cases and almost 10 million deaths from cancer were recorded worldwide. In addition, by 2040, the global cancer burden is expected to be more than 28 million cases [16]. This all confirms the need for new effective targeted treatment methods.

The identification of the processes and their biomarkers, which is responsible for initiation and enhanced tumor growth, is crucially important in the development of promising drugs for the treatment of oncological diseases. Over the past few years, many therapeutic targets have been identified for an effective cancer treatment [17,18,19,20]. In this review article, we have focused on the role of one of the leading determinants in the development and progression of oncological diseases—oxidative stress and inflammation. This expansion and systematization of the existing knowledge regarding the fight against cancer can help in the progression of drug development for cancer treatment. In addition, we summarize clinical and experimental data from the studies of perspective drugs of natural origin with the antitumor spectrum of action, aimed at oxidative stress and inflammation. These reviews can help both experimental scientists and clinical specialists. Moreover, they can facilitate the development of effective anti-cancer drugs and advance the research in this area, in order to improve the treatment of these disorders.

## 2. Role of Oxidative Stress in Cancer Progression

### 2.1. Free Radicals and Oxidative Stress—General Information

Recently, increased attention has been paid to the discussion of the free radical carcinogenesis theory. Despite the wide variety of pathogenetic pictures in each tumor type, there is a basic similar pattern of redox imbalance formation that leads to the oncopathology formation and progression [7,21,22]. Free radicals that cause genetic mutations can contribute to the initiation of the processes of normal cell transformation into a tumor cell, while simultaneously playing an essential role in all carcinogenesis stages [23,24].

The high reactivity of free radicals is due to the fact that, unlike common organic molecules, they have an unpaired electron in the outer electron orbital. In this regard, free radicals act as active oxidants, capturing the missing electron from various compounds and thereby, damaging their structure [25].

**Figure 1:**
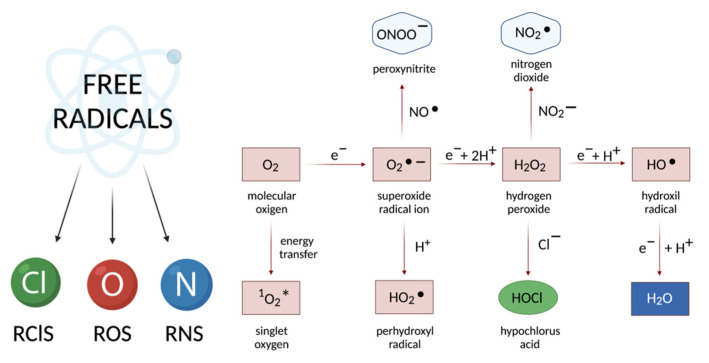
Scheme of free radical formation. The fundamental reaction for the formation of highly reactive free radicals is the conversion of molecular oxygen into water due to electron transfer. At different stages of this chain, the following can be formed: Reactive oxygen species (ROS), nitrogen (RNS), and chlorine (RClS).

This paper provides a conditional classification of free radicals, where they are divided according to the fundamental element into reactive forms of oxygen, nitrogen, and chlorine (Figure 1), while all types of free radicals can be combined and characterized as reactive, short-lived, interconverting forms of molecules, resulting from electronic excitation or redox transformations of the latter.

Oxidative stress is an imbalance between the formed reactive oxygen species and other highly reactive compounds and antioxidants, which leads to the disruption of redox processes and control and/or molecular damage with insufficient functioning of the antioxidant system [26,27]. Oxidative stress occurs as a result of ROS, RNS, and RClS overproduction, as well as a decrease in the cell antioxidant capacity. This is in turn accompanied by a number of negative effects due to the imbalance between the hyperproduction of free radicals and a decrease in the amount of antioxidant molecules [28]. ROS can be generated both by extracellular substances from environmental resources, in particular, chemical stresses, exposure to ultraviolet rays, ionizing radiation, and pollutants [29,30,31], and by intracellular sources, including mitochondria, peroxisomes, as well as immune system cells (i.e., neutrophils, eosinophils, and macrophages) [32]. Mitochondria are considered as the main intracellular source of free radicals due to the use of a significant amount of cellular oxygen (~90%) by these organelles, resulting in the formation of a significant number of short-lived intermediates, including hydrogen peroxide (H_2_O_2_), superoxide anion radical (O_2_^−^), and hydroxyl radical (OH) [33]. Therefore, free radicals of both enzymatic and non-enzymatic origin can cause oxidative damage to a wide range of biological macromolecules.

**Figure 2:**
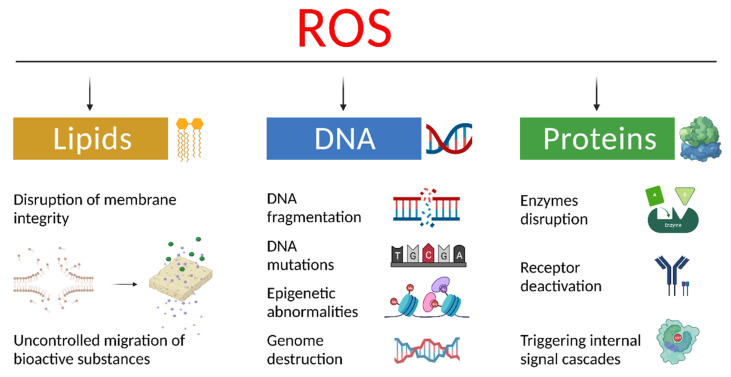
Consequences of the reactive oxygen species action in the cell during oxidative stress. Hyperproduction of reactive oxygen species disrupts the cell functioning by persistent oxidative damage to lipids, nucleic acids, and proteins due to the disruption of various links in the functioning of these macromolecules.

The cell membrane is one of the most vulnerable areas of ROS damage. Free radicals can react with polyunsaturated fatty acids of the cell membrane and form lipid peroxides. The accumulation of lipid peroxide can lead to the formation of carcinogenic agents, for example, malondialdehyde [34]. Damage to the cell membrane due to lipid peroxidation can permanently impair the membrane fluidity and elasticity, which leads to cell rupture. Proteins are another major target for free radical attack. Excessively produced radicals can react with the amino acids of proteins, which leads to oxidation and crosslinking. Radical protein reactions can permanently disrupt the function of important cellular and extracellular proteins, such as enzymes, various receptors, and connective tissue proteins [35,36]. In addition, DNA is very susceptible to the action of free radicals [37]. The interaction of an oxygen radical with DNA can lead to the impairment of its chains or the base removal. This DNA damage can be fatal to the body. Moreover, although the cell repair system corrects most of these damages, the DNA damage caused by radicals can be an important etiology of cancer development processes (Figure 2).

### 2.2. Oxidative Stress Biomarkers for the Determination of Oncopathology

The oxidative stress biomarkers, which may serve as oncopathology identifiers, can all be divided into three large conditional groups: (1) End products of biomaterial oxidation (lipids, proteins, DNA); (2) genes encoding various enzyme proteins and related to the redox balance; and (3) the quantitative level of these enzymes and their activity.

**Figure 3:**
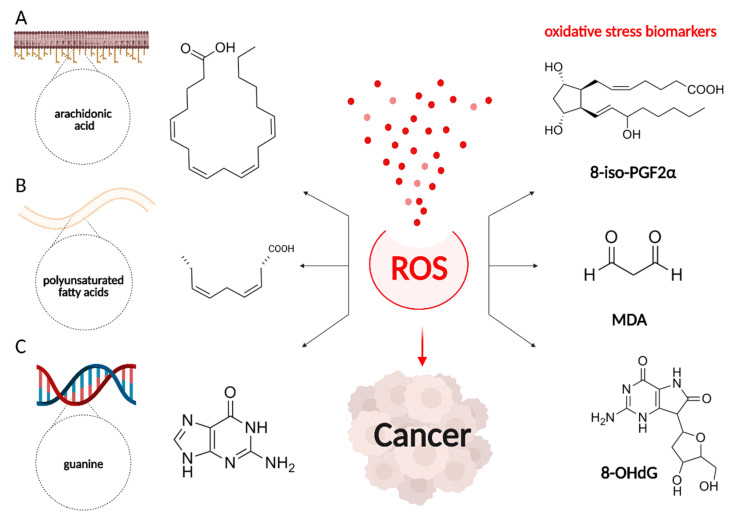
A schematic representation of the formation of key oxidative stress biomarkers, in which the increased level of urine or blood serum may be a sign of the formation and progression of malignant neoplasms. The steady preservation of excessive amounts of reactive oxygen species leads to aberrant oxidation of (**A**) arachidonic acid present in phospholipid membranes, resulting in the formation of 8-iso-prostaglandine F2α (8-iso-PGF2α); (**B**) polyunsaturated fatty acids with the formation of malondialdehyde (MDA); and (**C**) intracellular compound and DNA component guanine, which leads to the formation of 8-hydroxy-2′-deoxyguanosine (8-OHdG).

Numerous studies confirm that the excessive ROS production contributes to the progression of carcinogenesis and neoplasms [38,39]. In the case of cancer dynamics, it has been shown that it is directly related to the hyperproduction of lipid peroxidation products, as well as DNA-structurally diverse compounds, which can be found in body fluids. The most common markers for the determination of pathology are malondialdehyde (MDA) and 8-hydroxy-2′-deoxyguanosine (8-OHdG), found in the blood of patients, and 8-isoprostaglandin F2α (8-iso-PGF2α), found in urine and blood (Figure 3) [38,40,41].

Patients with various cancer forms and types were characterized with an increased MDA level, the final product of membrane phospholipids peroxidation in blood serum compared with the control group [42]. The increase in MDA level was shown in patients with breast cancer compared with healthy controls [43,44,45], which is associated with excessive ROS production and deficiency of the inherent antioxidant defense system. The LPO level significantly increased in patients with stage III and IV breast cancer [46,47], and the activity of GPx and SOD enzymes in serum samples of breast cancer patients decreased compared with healthy controls [45].

The evaluation showed that the MDA levels in lung cancer patients also progressively increased as the disease progressed, especially in stages III and IV. The activity of antioxidant defense system enzymes was significantly reduced in patients with the disease [48], while patients with prostate cancer had increased MDA levels compared with healthy people [49] and patients with benign prostatic hyperplasia [50]. Regarding kidney and bladder cancer, the serum MDA levels were significantly higher in both cancer types, but with no correlation with the stage of the disease [51,52]. The LPO levels and antioxidant status were also measured in patients with oral and oropharyngeal cancer, where the increase in MDA levels and the decrease in the amount of antioxidants in blood plasma were detected [53]. Interestingly, both an increase [54,55] and a decrease in MDA levels [56] have been shown in patients with gastric cancer.

A higher oxidative stress level and a lower antioxidant protection level also correlate with the progression of colorectal cancer, as evidenced by the significant increase in MDA level along with the decrease in the level of antioxidants (vitamins E and C) [57,58]. The LPO levels were also significantly higher in patients with liver cancer compared with healthy controls, while the levels of antioxidant enzymes and exogenous antioxidants were significantly lower [59]. A similar pattern was observed in patients with ovarian cancer, which is believed to be due to excessive ovulation or epithelial inflammation [60]. In addition, it was found that the oxidative stress levels are higher in patients with stage IV ovarian cancer than in patients with stage II. In patients with stage II and higher cervical cancer, the higher lipid peroxide levels were found in the blood serum compared with healthy people [61].

Another biomarker reflecting the oxidative stress level is 8-iso-prostaglandin F2α (8-iso-PGF2α), which is formed as a result of free radical-mediated arachidonic acid oxidation. It is stable in both urine and blood samples and is available for detection with reliable quantification methods. Szymańska et al. has found that 8-iso-PGF2α median in urine was 1.5 times higher in patients with bladder cancer than in the control group. However, there was no correlation between the 8-iso-PGF2α level and the degree of malignancy and invasiveness of this disease [62]. Zhang L.J. et al. using tandem mass spectrometry with ultra-high-performance liquid chromatography (UPLC-MS/MS) found that in the urine and blood serum of patients with colorectal cancer, compared with healthy volunteers, abnormal levels of polyunsaturated fatty acid metabolites were observed, in particular, 2,3-dinor-8-iso PGF2α, 19-HETE, and 12-Keto-LTB4 [63]. In addition, the experimental study on the murine model of colitis-associated colon cancer showed that in urine collected precisely in the carcinogenesis phase, and not in the acute colitis phase, there is a significant increase in F2-Isoprostanes (F2-IsoPs), 8-iso-PGF2α, and 2.3-dinor-8-iso PGF2α levels compared with healthy phenotype animals. The morphological examination showed that infiltrated monocytes in the tumor mass strongly expressed the NADPH ROS generator (p22phox). These observations suggest that 8-iso-PGF2α and 2.3-dinor-8-iso PGF2α in urine may be indices of colorectal cancer [64].

Another important biomarker of oxidative stress and carcinogenesis is 8-OH-deoxyguanosine (8-OHdG), a modified nucleoside formed in the DNA molecule as a result of exposure to reactive oxygen species and other damaging factors [65]. Unlike other modified oxidized guanine forms, 8-OHdG easily penetrates from the cells into the bloodstream. As a result, it is considered one of the best clinical and laboratory markers, in which its level may be used to evaluate an existing pathology or predict its early development.

One study has suggested that 8-OHdG is a prognostic factor for epithelial ovarian carcinoma. High 8-OHdG levels are associated with poor survival in ovarian cancer, which correlates with traditional factors of poor prognosis and serous histology. The serum concentration of 8-OHdG was also noticeably higher in stage III–IV carcinomas compared with more localized ovarian tumors. The increased 8-OHdG levels were found in high-grade papillary-serous carcinoma, but neither in low-grade papillary-serous carcinoma nor in cystadenoma [66]. Significantly higher 8-OHdG values were shown in patients with chronic atrophic gastritis and gastric carcinoma [56]. Similarly to the above, the 8-OHdG imbalance indicated an increased risk of breast cancer in postmenopausal women [67]. Moreover, it was shown that the average 8-OHdG level in three groups of patients with colorectal carcinoma (adenoma, early cancer, and advanced cancer) was significantly increased, which suggested that the increase in 8-OHdG concentration in the blood was a risk factor for colorectal adenoma and early cancer [68]. Furthermore, the increase in 8-OHdG level was observed in patients with prostate [69], esophageal [70], as well as head and [71,72] cancer types.

Table 1 briefly presents the biomarkers that belong to the second and third groups, which are genetic or enzymatic in nature.

The authors of [99,100] have shown that the high NOX4 expression in patients with gastric cancer correlated with worse overall survival, especially in patients with intestinal tumors. NOX4 is also highly expressed in non-small cell lung cancer (NSCLC) and promotes cancer progression by inducing glycolysis during c-Myc activation via the ROS/PI3K/Akt pathway, while GKT137831, a selective NOX4 inhibitor, suppresses the growth of tumor cells both in vitro and in vivo [101]. The high NOX4 expression predicted the worst clinical outcome in terms of overall survival in patients with endometrial [102] and ovarian cancer [103]. In addition, the knockdown of this gene in ovarian cancer cells has increased the sensitivity to chemo- and radiotherapy, which suggests a key role of NOX4 in the development of drug resistance. The treatment of urothelial bladder carcinoma cell lines overexpressing NOX4 with diphenylene iodonium significantly reduced the level of intracellular ROS and induced cell cycle arrest in the G1 phase. Moreover, the NOX4 blockade with siRNA suppressed the growth of cancer cells in an in vivo mouse orthotopic model [104].

The analysis of GPx and SOD activity in head and neck tumor tissue samples showed significantly lower levels of antioxidant enzymes in low-grade tumors [105]. A similar situation was found in patients with gastric cancer [106], oropharyngeal squamous cell carcinoma [107], prostate cancer [108], bladder cancer [109,110], ovarian cancer [111], etc. Similar to superoxide dismutase, the reduced CAT and GPx expression and activity compared with healthy cells was observed in colorectal cancer [112]. The author of [113] found that in patients with lung cancer, the catalase activity of erythrocytes was reduced and significantly decreased in patients with metastases. Interestingly, the decrease in the proliferation of A549 cells exposed to curcumin was accompanied with the increase in CAT and SOD activity and the decrease in ROS levels [114]. The high GPx1 expression was also found to be associated with a poor overall survival prognosis in brain lower grade glioma [115], acute myeloid leukemia [116], and bladder cancer [110].

Another oxidative stress predictor is an inducible nitric oxide synthase (iNOS), which generates nitric oxide (NO). Nitric oxide hyperproduction was shown to increase the resistance of triple-negative MDA-MB-231 cells to cisplatin, while triple-negative breast cancer patients with reduced iNOS levels in tumor cells after the treatment showed a better response to platinum-based neoadjuvant chemotherapy [117]. The authors of [118] found a positive correlation between the high iNOS expression and TNM staging of breast cancer. The increased iNOS activity was also found in tissue samples of colorectal cancer [119], which plays a crucial role in the angiogenesis of this type of neoplasm [120], in patients with bladder cancer [121], who are accompanied with high NO levels in tumor tissues, urine, and blood serum [52,122], with pancreatic cancer [123], non-small-cell lung carcinoma [124], as well with head and neck squamous carcinoma [125], glioblastoma [126], and melanoma [127]. In addition, it is associated with a poor prognosis in patients. Recently, it has been shown that in colon- [128], lung- [129], and breast cancer [130], NO also regulates the growth and aggressiveness of cancer stem cells.

The enzymes involved in antioxidant protection are also variously localized paraoxonases (PONs), including plasma, plasma membranes, endoplasmic reticulum, nuclear envelope, and inner mitochondrial membrane [98]. The decrease in PON1 activity was found in the blood serum of patients with genital tract neoplasms, including endometrial cancer [131], with colorectal [132] and bladder [133] cancers, while the lower PON1 concentration was observed in patients with tumor recurrence compared with patients without relapse [134]. At the same time, the studies showed the increased PON2 expression in non-melanoma skin cancers in basal cell carcinoma cells, which had a positive correlation with metastasis to lymph nodes, parameters, and the pathological stage of the tumor [135]. The increase in PON2 expression was also found in tissue samples obtained from patients with gastric cancer, which had a significant positive correlation with the diffuse type, clinical stage, tumor invasion, lymph node metastases, and distant metastases. Moreover, the survival analysis showed that the aberrant PON2 expression led to a significantly shorter overall survival compared with patients with a low expression of this gene [136]. Similar results were obtained in the study of PON2 expression in bladder cancer [137], oral squamous cell carcinomas [138], and glioblastoma multiforme [139].

**Figure 4:**
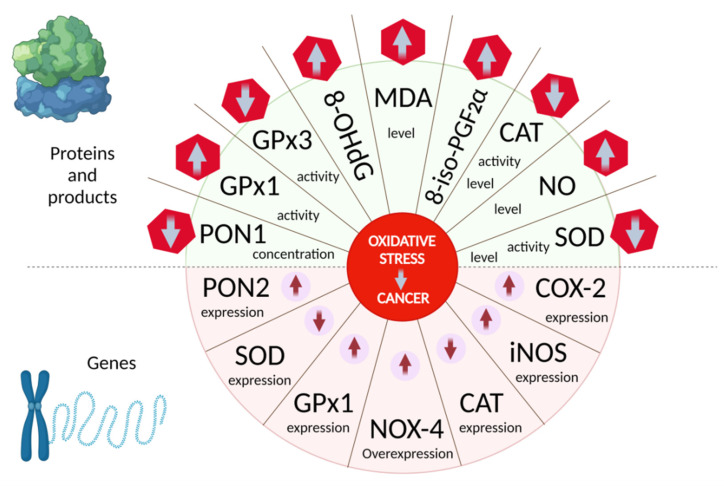
Schematic diagram reflecting the main commonly measured biomarkers of oxidative stress, impaired level, expression, and activity, which have a prognostic value in cancer progression.

The generalized data on changes in the levels of gene expression and the activity of the corresponding enzymes, as well as the concentration of the final oxidation products in cancer conditions are shown in Figure 4.

To summarize, we can say that the previously described disorders are of both endogenous and exogenous origin, which leads to the overproduction of free radicals. As a consequence, cellular oxidative stress is of great importance in the development of malignant neoplasms and can be considered as a key biomarker of cancer development and progression.

### 2.3. ROS-Induced Pro-Oncogenic Signaling

**Figure 5:**
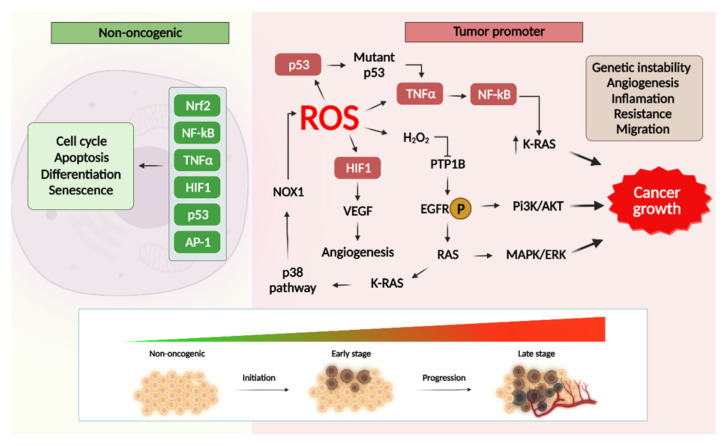
Activation of transcription factors and triggering of pro-oncogenic signaling pathways under the ROS action in the context of oxidative stress conditions. Transcription factors, such as AP-1, NF-κB, HIF1, TNFα, p53, etc. normally regulate important processes of vital activity in the cell (differentiation, aging, and apoptosis). In the case of oxidative stress conditions, when the excessive accumulation of free radicals occurs, ROS activate certain signaling cascades including PI3K/AKT and RAS-MEK-ERK pathways, which leads to genetic cell instability, excessive angiogenesis, inflammation, and uncontrolled proliferation, as well as trigger the process of transformation of a healthy cell into a tumor.

Numerous studies confirm that ROS and oxidation products control oncogenesis in cells through pro-oncogenic signaling pathways [140]. In Figure 5, it has been shown that due to the activation of a number of signaling factors, in particular, p53, TNFα, NF-κB, HIF1α, VEGF, etc., the reactive oxygen species contribute to the activation of downstream pathways phosphatidylinositol-3-kinase/protein kinase B (PI3K/AKT) and RAS/MEK/ERK (ERK/MAPK), which leads to the transformation of a healthy cell into a tumor, as well as cell survival and hyperproliferation, avoidance of apoptosis, invasion, metastasis, and angiogenesis [140,141,142]. In turn, Ras, as a GTPase, is often involved in oncogenesis by activating MAPK pathways and regulating transcription. The previously mentioned pathways all prevail in the cancer development. For instance, inhibition of the MAPK/ERK and PI3K/AKT pathways through the ROS-dependent pathway leads to the death of BRAF wild-type thyroid cancer cells [143].

Moreover, ROS as signal messengers can activate important target molecules involved in gene transcription pathways, which are capable of regulating the redox status of cells and influencing the transformation of a normal cell into a tumor cell, the neoplasm growth, and hyperproliferation. These transcription factors include Nrf2, AP-1, NF-κB, HIF1, TNFα, and p53 [144,145,146,147,148].

The Nrf2-Keap1 pathway is the major regulator of cytoprotective responses to endogenous and exogenous stresses caused by reactive oxygen species (ROS). Nrf2 is a transcription factor that can bind to the antioxidant response element (ARE), which leads to the expression of antioxidant response genes [149,150]. At normal low physiological ROS levels, Nrf2 is poorly expressed. As soon as the level of free radicals increases by stimulating the expression of the corresponding gene, conformational changes of the Nrf2 molecule occur due to the separation from the Nrf2-Keap1 complex. This mainly allows the translocation of the cytoplasmic protein Nrf2 into the nucleus and direct binding to the ARE, which leads to the increased activation of the gene [151]. The Nrf2-Keap1 complex serves as a cellular defense mechanism that responds to cellular stress from endogenous and exogenous agents [152].

**Figure 6:**
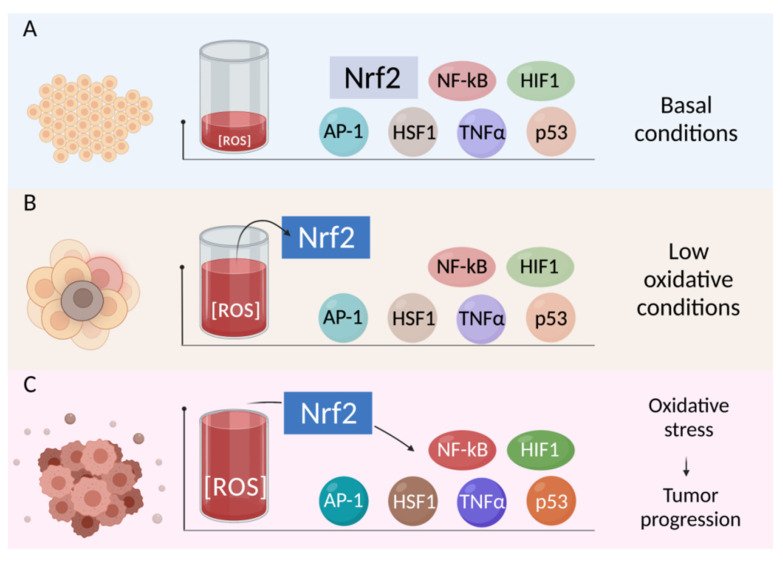
The role of Nrf2 in protecting the cell from oxidative stress. Under stress-free baseline conditions (**A**), the level of reactive oxygen species is maintained at a consistently low level due to the activity of antioxidants. However, with increased oxidative stress (**B**), Nrf2 activation, which ensures the adaptation of cells to an increase in ROS, as well as the induction of several genes that encode detoxification enzymes and antioxidants occur. If the ability of antioxidant systems induced by Nrf2 becomes insufficient to counteract additional oxidative stress (**C**) or oxidative stress proceeds for a long time, N2 suppression occurs. In addition, excessive ROS levels, which are not counteracted by Nrf2-directed protection, trigger additional redox triggers that activate other antioxidant transcription factors, which leads to various cellular responses, including metabolic reprogramming, damage repair, avoidance of apoptosis, and other features typical of tumor cells.

ROS, RNS, and lipid aldehydes have been shown to form as a result of exposure to toxic substances, which leads to the activation of Nrf2, and this may be associated with cancer induction [153]. Moreover, Nrf2 plays an important role in regulating the expression of antioxidant enzymes, including glutathione reductase, catalase, superoxide dismutase, and glutathione peroxidase. Consequently, low levels of Nrf2 expression can indirectly lead to an increase in ROS formation and, therefore, DNA damage through a decrease in the normal antioxidant cell capacity [154]. Although Nrf2 was initially recognized as a target of chemotherapeutic compounds with the ability to activate this transcription factor [155,156], to date, a large amount of data have been accumulated that show that Nrf2 is the driving force of cancer progression, metastasis, and resistance of tumor cells to the treatment [157,158,159,160]. The authors of [161] have shown that Nrf2 can accelerate the proliferation of cancer cells by directing anabolic purine synthesis pathways. This makes the cells more susceptible to tumor initiation and transformation, and provides the tumor cells with advantages in survival and growth. Moreover, it is assumed that the expression of the gene directed at Nrf2 provides the initial means of adaptation to oxidative stress. At later redox imbalance stages, due to its translocation into the nucleus, Nrf2 triggers the expression of a battery of antioxidant genes and activates other antioxidant transcription factors in the network [144,162] (Figure 6).

Activator protein 1 (AP-1) is another transcription factor that has been shown to be activated by oxidative stress and changes in the intracellular redox status. In addition, it includes various members of the Jun, Maf, Fos, and ATF families, which play an important role in differentiation, proliferation, and apoptosis [163]. Changes in the expression of AP-1 components are recorded in various malignant neoplasms, in particular, in samples of patients with breast carcinoma, gynecological cancers, gastrointestinal carcinomas, and hematological malignancies (in chronic myelogenous leukemia and acute myeloid leukemia, Hodgkin’s lymphoma and anaplastic large cell lymphoma) [164]. For instance, increased levels of Jun, JunB, Fos, and FosB mRNA are recorded in the biopsy material obtained from patients with inflammatory breast cancer, in comparison with healthy tissue samples [165]. At the same time, a decrease in JunB expression was shown in the peripheral blood of patients with chronic myelogenous leukemia [166,167], which indicates the prospects of considering AP-1 as a marker of the progression of malignant neoplasms.

NF-κB is a nuclear transcription factor that participates in a number of normal cellular and tissue processes, including cell survival and differentiation, as well as the modulation of the inflammatory response [168]. Normally, NF-κB exists in a dimeric inactive form, while its activation occurs due to the effects of stress factors, including oxidative stress. The activation of NF-κB is closely related to the process of carcinogenesis, and its functional consequences correlate with the development and progression of malignant neoplasms. In addition, this NF-κB activity is proven to be a critical mechanism, contributing to tumorigenic processes in pancreatic cancer [169], lung [170], breast [171], cervical [172], gastric [173], and prostate cancer [174]. The important role of NF-κB in the cancer development is shown by the following factors: (1) The ability to change the metabolism of tumor cells from mitochondrial-dependent oxidative phosphorylation to anaerobic glycolysis, which leads to the emergence of the Warburg effect and the adaptation of cancer cells to hypoxia conditions [175,176]; (2) the assurance that the transformed cells avoid death by enhancing the expression of antiapoptotic genes; (3) increased proliferation of cancer cells through the regulation of expression of cyclins and proto-oncogenes; as well as (4) the promotion of metastasis and angiogenesis [177]. In addition, all of these factors make NF-κB a promising therapeutic target for the creation of chemotherapeutic agents for the treatment of malignant neoplasms [178].

HIF1 is a constitutively expressed transcription factor [179] and cell concentration, which is strictly controlled by the oxygen level. In normal cells, HIF1α is rapidly destroyed after formation, while in hypoxic cells, the HIF-1α subunit is not hydroxylated and thus forms a stable complex with HIF-1β. HIF1 is involved in ROS-induced carcinogenesis in a number of human tumors [180], and its high concentration, especially HIF-1α, was closely related to the aggressive behavior of tumors and correlated with poor patient survival outcomes [181]. Significant associations between the HIF1 overexpression and patient mortality have been shown in various cancer types, including breast [182,183] and cervix cancer [184,185], non-small cell lung cancer [186], sarcoma [187], etc. Moreover, HIF1 activation increases the expression of proangiogenic genes, for example, the vascular endothelial growth factor, which is associated with metastatic disease [188].

P53, a tumor suppressor, has also been shown to play an important role in the antioxidant response. Its mutant form may activate TNFα and trigger a cascade of activation processes of other transcription factors, which leads to tumor transformation [189].

Therefore, it is possible to declare that oxidative stress plays an important role in the origin and progression of various cancer types. By analyzing the data on numerous links of tumor pathogenesis that are associated with the processes of redox imbalance, it is possible to obtain important information on potential therapeutic targets for the search for multi-targeted effective antitumor agents. Moreover, summing up the knowledge on the changes occurring in various cancer types with representatives of the antioxidant defense system and the levels of the end lipid, protein, and DNA oxidation products, it is possible to identify and predict cancer severity by analyzing these components in the blood serum or urine.

## 3. Role of Inflammation in Cancer Progression

At the start of the XIX century, a relationship between cancer and the inflammatory cascade has been suggested based on observations that inflammatory cells were present in biopsy tumor samples, which often occurred in chronic inflammation sites [190]. At a later time, this hypothesis was confirmed by the results of several studies, ranging from epidemiological studies of patients to molecular studies of genetically engineered mice [191,192,193,194,195].

**Figure 7:**
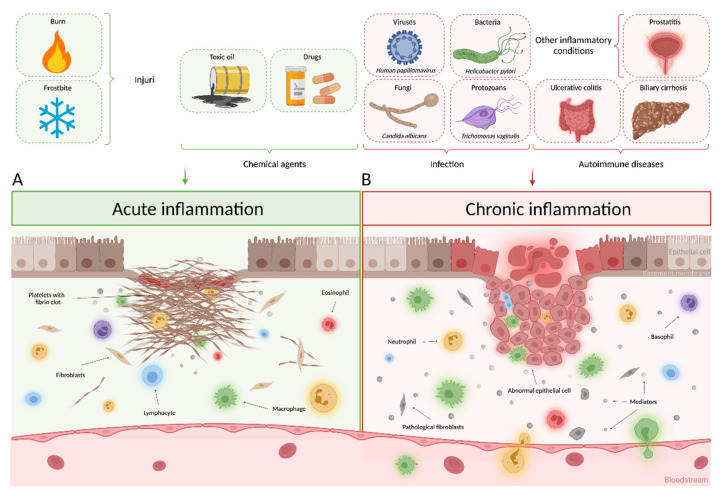
Comparison of wound healing in acute inflammation and the neoplasm development in chronic inflammation. (**A**) Acute inflammation is caused by stress, such as tissue damage or surgery, as well as by the action of various chemical agents. Normally, when tissues are damaged, platelets are activated, which forms a hemostatic plug and releases mediators that regulate vascular permeability and the formation of a fibrin clot; the venous network is restored, and repeated epithelialization occurs throughout the wound, which promotes healing and termination of the complex process of interaction of immune, stromal, and epithelial cells. An untimely stop of acute inflammation leads to its transition to a chronic form. (**B**) Chronic inflammation can occur for various reasons (continuous contact with infection, autoimmune diseases, and other inflammatory conditions) and can be a trigger for the formation and progression of cancer diseases. Hyperactivated cells of the immune system (macrophages, neutrophils, lymphocytes, basophils, and eosinophils) secrete a variety of inflammatory mediators (cytokines and chemokines). In turn, these systemic increases of mediators are a trigger for the transformation of normal cells into abnormal cells, which enhance tumor growth in malignant neoplasms, stimulate angiogenesis, and induce migration and maturation of pathological fibroblasts, which contribute to the spread of metastases.

Inflammation is one of the most fundamental and pronounced protective body reactions, which includes a long chain of molecular reactions and cellular activity. In addition, it occurs in response to tissue damage caused by physical trauma, ischemic injury, infection, exposure to toxins or other types of injuries [196,197]. The inflammatory body reaction causes cellular changes and immune reactions that lead to the recovery of the damaged tissue and cell proliferation at the site of its damage [198]. If the cause of inflammation persists or certain control mechanisms that are responsible for stopping the process fail, the inflammation becomes chronic [199], which leads to mutations, in particular, that contribute to the development of cancer [200,201,202]. Even though many of the molecular mediators are equally formed in both acute and chronic inflammation, acute inflammation is not considered as a risk factor for the neoplasia development (Figure 7).

**Figure 8:**
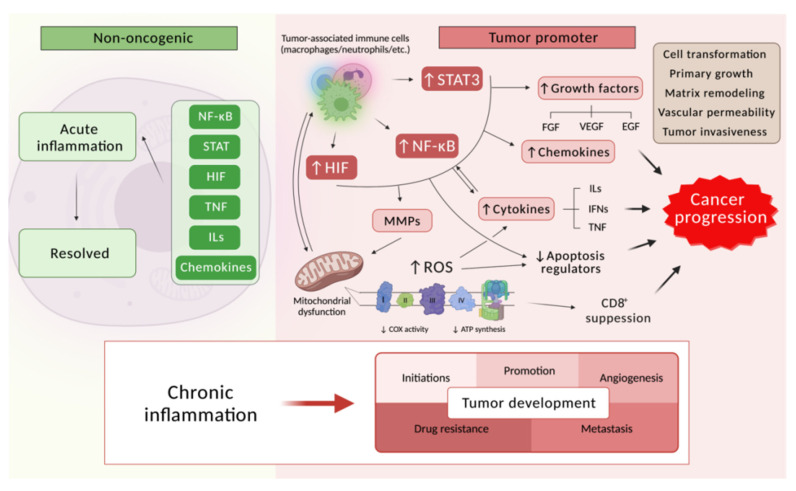
Non-oncogenic inflammation pathway and tumor-promoting inflammation. In the case of the acute phase of inflammation, inflammation is activated which occurs due to pro- and then anti-inflammatory modulators. If acute inflammation is not eliminated, pathologies with chronic inflammation develop. Chronic inflammatory or infectious conditions mediate the risk of cancer development and progression due to the activation by tumor-associated immune cells of transcription factors, mainly nuclear factor NF-κB, signal transducer and activator of transcription 3 (STAT3), and hypoxia-inducible factor (HIF1) in cells. At the same time, transcription factors coordinate the production of growth factors and various enzymes, as well as inflammatory mediators, including chemokines and cytokines, which can in turn activate the same transcription factors in inflammatory, stromal, and tumor cells, contributing to the formation of cancer-related inflammatory microenvironment. Chronic inflammation leads to mitochondrial dysfunction and a decrease in ATP synthesis, causing hyperproduction of reactive oxygen species that inhibit the activity of apoptosis regulators and CD^8+^ cytotoxic lymphocytes. All of these events that occur in chronic inflammation contribute to cancer progression and participate in all of the major stages of tumor development, from nucleation to metastasis and the formation of drug resistance.

In recent years, a significant amount of data have accumulated, indicating that many malignant neoplasms arise as a result of prolonged infection, and as a consequence of a chronic inflammatory process that forms the tumor microenvironment through various pathways [203]. Numerous triggers of chronic inflammation increase the risk of malignancy development and progression (Figure 7), including (1) infections of various origins, for example, the main risk factor for stomach cancer and mucosa-associated lymphoma is bacterium Helicobacter pylori, which leads to a significant formation of nitric oxide that damages the host nucleotide DNA and alters the regulation of transcription through the activity of DNA methyltransferase [204,205,206,207], the Candida fungi increase the risk of hematological malignancies, cancers of the oral cavity, lips, pancreas, skin, and thyroid gland [208,209,210], an infection with Trichomonas vaginalis correlates with an increase in the cervical cancer incidence [211] and prostate cancer [212], and cells infected with Human papillomavirus that release various cytokines create an inflammatory environment, which leads to the activation of pro-oncogenic signaling pathways that contribute to the development of cervical carcinoma [213,214,215,216,217]; (2) autoimmune diseases—inflammatory bowel diseases, such as Crohn’s disease and ulcerative colitis are associated with an increased risk of intestinal adenocarcinoma [218,219,220,221]; and (3) inflammatory conditions of uncertain origin, for example, prostatitis in prostate cancer [222,223]. Chronic inflammation has been proven to be associated with various stages of tumor formation, including cellular transformation, promotion, survival, proliferation, invasion, angiogenesis, metastasis, and drug resistance (Figure 8) [224,225].

The tumor microenvironment, as known, consists of dividing tumor cells, stroma, blood vessels, and various inflammatory cells that are widely involved in oncogenesis [226]. The inflammatory neoplasm component may include a population of leukocytes, neutrophils, dendritic cells, macrophages, myeloid derived suppressor cells, eosinophils, and natural killer cells that are capable of producing a wide range of signaling molecules, in particular, cytokines, cytotoxic mediators, including reactive oxygen species, proteolytic enzymes, etc. (Figure 8).

Macrophages play a key role in the process of chronic inflammation, as an important component of inflammatory infiltrates in neoplastic tissues [227]. Macrophages are differentiated cells of myeloid origin, abundantly present in most microenvironments of malignant tumors [228]. Tumor-associated macrophages (TAMs) may play a dual role in neoplasms: They can both kill tumor cells after activation by IL-2, interferon, and IL-12 [229]. In addition, they perform a variety of functions related to cancer development and progression: They promote the release of tumor cells into the circulatory system and suppress antitumor immune mechanisms and responses [230]. A number of studies have shown that macrophages can promote extravasation and continuous growth of circulating cancer cells in remote locations, which is a key factor in the growth of metastatic colonies [231,232,233,234,235]. Moreover, a large amount of data indicate the important role of macrophages in the formation of resistance in tumor cells to the radio and chemotherapeutic treatment [236,237,238]. In experimental models, it was found that when the number of macrophages is depleted, tumor progression is inhibited, which indicates their important role in mediating tumor progression events in the induced transgenic mouse model of precancerous progression [239].

Upon activation, there are two main macrophage phenotypes, classic M1 and alternative M2 [240], in which various inflammatory mediators are formed, that play an important role in tissue remodeling [241]. Tumor-associated macrophages usually refer to an alternative M2 phenotype with different subsets. In addition, they represent universal and multitasking cells that directly affect several stages of tumor development through critical interactions with cells associated with tumor progression, such as T-regulatory cells (suppressor T cells), cancer stem cells, T-helper cells, cancer-associated fibroblasts, and myeloid-derived suppressor cells (MDSC) [242]. Moreover, the second type of macrophages has negative cross-contacts with tumor suppressor cells, including cytotoxic T cells and natural killer (NK) cells [243] and can neutralize the effects of the antitumor phenotype M1 [244], expressing high levels of molecules of the main histocompatibility complex responsible for the death of cancer cells [245,246].

TAMs affect many different aspects of the tumor cell behavior by producing growth factors, such as epidermal growth factor (EGF), fibroblast growth factors (FGF), vascular endothelial growth factor (VEGF) [247], as well as due to the production of cytokines capable of activating pathways that control apoptosis and cell proliferation through the modulation of certain genes. For instance, IL-6 activates STAT3, which controls cell survival by acting on various factors, such as cyclin D, B-cell lymphoma-extra-large (Bcl-XL), B-cell lymphoma 2 (Bcl-2), and induced myeloid leukemia cell differentiation protein (Mcl-1) [248,249]. In the hypoxic microenvironment of growing tumors, macrophages secrete a number of proangiogenic factors and produce several proteolytic enzymes and other mediators (for example, MMPs and cathepsins), which are involved in the remodeling of the extracellular matrix, the degradation that promotes the tumor cell penetration into blood vessels and the metastases spread [250].

Neutrophils are also one of the most common immune cells that are involved in inflammatory reactions in oncopathologies. Neutrophils play both a pro- and anti-tumor role, while with the development of a tumor, the number of neutrophils increases, eventually amounting to 90% of leukocytes [251]. Tumor-associated neutrophils (TANs) have been shown to have the ability to switch their phenotype from supportive to cytotoxic in mouse models. Depending on the tumor stage and the function performed, tumor-associated neutrophils are divided into three subgroups [252]: (1) High-density neutrophils and (2) low-density neutrophils with an antitumor effect, as well as (3) granulocytic-myeloid derived suppressor cells (G-MDSC), which due to the production of reactive oxygen species inhibit the activity of cytotoxic CD^8+^ lymphocytes, promoting tumor growth and progression (Figure 8) [253].

In addition to macrophages and neutrophils, the mast cells, eosinophils, and activated T-lymphocytes also play an important role in the potentiation of tumor processes, which contribute to malignant neoplasms by releasing extracellular proteases, proangiogenic factors, and chemokines [254,255,256]. In other words, inflammatory cells contribute to the destruction of the basement membrane, remodeling of the extracellular matrix, and cancer cell migration through the secretion of inflammatory mediators [224]. Interestingly, some of these cells induce the production of ROS and NOS in non-phagocytic cells by binding to specific receptors.

Tumor necrosis factor α (TNFα) is a multifunctional inflammatory cytokine that is secreted mainly by activated macrophages and plays a crucial role in many processes of functioning of both normal and malignant cells, including survival, apoptosis, and necroptosis, as well as intercellular communication [257]. A violation of the regulation of this complex signaling network is a distinctive feature of a wide range of inflammatory diseases [258], in particular, oncological pathologies [259,260,261,262]. TNFα functions by binding to two receptors (TNFR1 and TNFR2 receptor), which leads to the regulation of a number of cytokines, proteases, and growth factor production [263]. Both the tumor and stromal cells of solid tumors secrete TNFα. Interestingly, TNFα is able both to stimulate and inhibit tumor growth [259]. For instance, in patients with breast cancer, high levels of this cytokine are found both at the tumor site and in blood, which correlates with the invasive/malignant phenotype of the tumor and contributes to the development of this pathology in all stages, including the development of the primary tumor, epithelial mesenchymal transition, metastasis, and disease recurrence. Evidently, this is due to the ability of TNFα to trigger specific pro-oncogenic signaling pathways in the transformed cells [259]. The correlation of high levels of TNFα with the stage of clinical disease of breast cancer and metastases in the lymph nodes, as well as with the expression of antigens estrogen receptors and human epidermal growth factor receptor 2, is also described in [260]. Recently, Yoshimatsu et al. showed that TNFα enhances TGF-β-dependent endothelial-to-mesenchymal transition [264] and promotes cell development hepatocellular carcinoma [265]. In turn, the antitumor mechanism of TNFα lies in its targeting of the tumor-associated vasculature, causing increased permeability and destruction of the choroid, and as a consequence, leads to the selective accumulation of cytostatic drugs inside the tumor [266]. Therefore, it was shown that the addition of TNFα can modulate the activity of doxorubicin and lead to a significant increase in its content in the tumor tissue in models of sarcoma, as well as the effective regression of fibrosarcoma BN175 and osteosarcoma ROS-1 [267]. The enhancement of the antitumor effect of doxorubicin by TNFα, due to the suppression of the antiapoptotic activity of p21, was also confirmed by Jiang et al. [268], an in vivo bearing hepatoma H22 and sarcoma S180 allografted tumors [266].

TNFα has also been shown to induce the ROS production by neutrophils [269] in many cell types [270,271,272]. In addition, TNFα knockout mice demonstrate a significant decrease in the development of skin tumors in response to the treatment with 1,3-dimethylbutylamine (DMBA) [273]. In a study by Moon et al. [274], it was found that the treatment of human leukemia cells U937 by rosmarinic acid significantly sensitizes TNFα-induced apoptosis, which is accompanied by the suppression of nuclear transcription factor NF-κB and a decrease in the production of reactive oxygen species. The similar effect was shown in the investigation of the cell death profile of prostate cancer cells LNCap under arbutin action, which dose-dependently reduces the expression of TNFα and intracellular ROS when using tert-butyl hydroperoxide (TBHP) as an ROS inducer [275].

Interleukins are the class of inflammatory molecules, in which the main ones are IL-1β, IL-6, and IL-10, that are produced and secreted by various types of cells. The production of IL-1β stimulates inflammation at an early stage, and in cancer, this cytokine is associated with a dedifferentiated and more aggressive disease. This cytokine functions by activating the vascular endothelium, causing the infiltration of tumor inflammatory cells. IL-6 is a pleiotropic cytokine that is immediately released by immune cells (in particular, monocytes and macrophages) in response to an infection or tissue damage, thereby playing an important role in the acute phase responses and immune reactions that protect the host. IL-6 has also been shown to be overexpressed by several types of tumor tissues and can play an important role in various aspects of tumor behavior, including apoptosis, proliferation of tumor growth cells, migration and invasion, angiogenesis, and metastasis [276]. In addition, it has been shown that the protection of cancer cells from therapy-mediated damage occurs by transmitting signals that promote survival, anti-apoptosis, and recovery processes. IL-10, an interleukin produced by almost all leukocytes, functions as a suppressor of inflammatory mediators and plays an antiangiogenic role, which leads to tumor proliferation and metastasis by enhancing Bcl-2 regulation and immunosuppression [277,278].

High levels of interleukins were found in the blood and tumor tissues in most cancers, in particular, breast cancer (IL-6, IL-8, and IL-1β) [260,279,280], colorectal cancer (IL-6, IL-17, and IL-34) [281,282,283], prostate (IL-6) [284] and ovarian cancer (IL-6, IL-8, and IL-33) [285,286,287], lung cancer (IL-6, IL-17, and IL-1β) [288,289,290], pancreatic (IL-6 and IL-18) [291,292] and cervical cancer (IL-6 and IL-10) [293,294], multiple myelomas (IL-6, IL-8, and IL-1β) [295,296], etc. This increase in interleukin levels is associated with aggressive tumor growth and the development of a resistance phenomenon in response to therapy [297,298,299]. An interesting factor is that at the end of the last century, it was shown that the treatment of glia and cultured smooth muscle cells of the rat colon with IL-1β led to a significant increase in the activity of inducible nitric oxide synthase (iNOS) [270]. Similarly to the above, it was found that a decrease in IL-1β and IL-6 levels, as well as the suppression of mRNA expression of genes encoding these cytokines, correlated with the lower NO release levels in RAW264.7 cells inflammation model under the action of anti-inflammatory fraction A [300]. IL-6 was also known to be activated by prostaglandin E2 (PGE2) that is formed during the reaction catalyzed by cyclooxygenase-2 [301], in which the elevated levels are observed in inflammatory macrophages in various types of cancer [75,302,303,304].

Chemokines are small molecules, which are secreted by a wide range of immune cells and may directly influence carcinogenesis and metastasis via modifying the tumor phenotype [305]. However, tumor cells not only regulate the expression of chemokines to recruit inflammatory cells, but also use these factors for further neoplasm growth and progression. For instance, it was found that the chemokines C-X-C motif ligand 1, 2, 3, and 8 (CXCL1, CXCL2, CXCL3, and CXCL8) are overexpressed in melanomas, which is accompanied by increased proliferation of tumor cells and tumor metastasis [306,307]. At the same time, blocking the receptors GROα, GROβ, GROY, and CXCR2, specifically associated with these chemokines, weakens these effects. The study results [308] showed high concentrations of chemokines CXCL1–2, CXCL4–5, CXCL7–10, CXCL12–14 in peripheral blood and blood drainage from tumors of patients with early gastric cancer. Interestingly, patients with low CXCR1 and CXCR3 expression had a smaller tumor volume and a lower TNM stage. However, with a decrease in CXCR2 and CXCR4, the opposite picture was observed. Paczek et al. and Lukaszewicz-Zajac et al. [309,310] showed that in the blood serum obtained from patients with colorectal and oesophageal cancers, the increase in the CXCL8 level was found, rising in the group of patients with distant metastases. The CXCL8 knockdown using an antisense vector led to the increase in cell death and the decrease in tumor growth in mouse models carrying xenografts [311]. A similar effect on CXCL13 was observed in patients with resistance to 5-fluorouracil [312]. It has also been shown that CXCL5 overexpression promotes tumor angiogenesis by activating the AKT/NF-κB/FOXD1/VEGF-A pathway [313], and correlates with lymph node metastasis and poor prognosis [314].

Transcription factors that modulate the synthesis of key inflammatory mediators, the recruitment of immune cells, and the functions of these cells in the tumor microenvironment also play an important role in cancer-related inflammation.

NF-κB is a family consisting of five transcription factors, involved in the innate immunity formation and in coordinating inflammatory responses [315]. NF-κB activation can be mediated both by signaling pathways of Toll-like receptor TLR-MYD88 and the previously mentioned inflammatory cytokines TNFα and IL-1β [316,317,318], as well as genetic changes in tumor cells [319]. In most cases of malignant neoplasms, NF-κB is constitutively active and plays a role in oncogenic transformation through aberrant activation of anti-apoptotic genes [320,321] and cell cycle progression, stimulating chronic inflammation, thereby contributing to tumor development [195]. In inflammatory and tumor cells, as well as cells at risk of transformation, NF-κB is a key regulator of the production of growth factors, inflammatory cytokines, chemokines, and angiogenic factors, by triggering the expression of their coding genes [322]. It has been shown, for example, that the deficiency of the TIR8 gene highly expressed in the intestinal mucosa and encoding the receptor of the same name, which is in turn an inhibitor of NF-κB, correlates with the increased susceptibility to intestinal inflammation and carcinogenesis [294,295]. The inhibition of the NF-κB signaling pathway showed significant suppression of macrophage activation and neutrophil exudation in a mouse peritonitis model [296], as well as a decrease in tumor growth in mice carrying A549 xenografts [297].

Signal transducers and activators of transcription 3 (STAT3) is another cell signal transcription factor that plays an important role in the regulation of the antitumor immune response and, as a consequence, these processes of differentiation, proliferation, survival, angiogenesis, and invasion [323,324]. Hyperactivation of STAT3 is prevalent in both cancer cells and immune cells of the tumor ecosystem of various origins [325,326,327]. Moreover, STAT3 can be a convergence point for several oncogenic signaling pathways [328,329]. More importantly, aberrant STAT3 activation plays a key role in tumor progression, in the development of resistance to therapy, and relapses [330,331].

Hypoxia-inducible factors are a family of transcription factors that are the main regulators of the cellular response to hypoxia and coordinate the transcription program that ensures optimal functional, metabolic, and vascular adaptation to oxygen deficiency [332]. The family includes a number of representatives, in which the most known are HIF-1α. This is widely expressed and found in almost all populations of innate and adaptive immunity, and HIF2α, in which the expression is observed in a number of cell types, including endothelial cells associated with tumor macrophages [333,334], etc. It is well known that hypoxia is a typical feature of solid tumors [335] and leads to the HIF1α stabilization in cancer cells, promoting the recruitment of myeloid cells in two ways: Activating chemokine receptors and stimulating the chemokine production, as well as increasing the cytokine secretion. HIF1α removal in macrophages has been shown to lead to a decrease in tumor growth in the model of spontaneous breast adenocarcinoma PYMT, which is the result of an improvement in the proliferative ability and function of lymphocytes in the absence of myeloid HIF1α [336,337]. HIF-1α signaling in primary breast tumors leads to the induction of members of the lysyl oxidase family and to the metastatic colonization of lung cells [338]. For HIF2α knockout mice, there was a decrease in infiltration, migration, and TAMs expression in mouse models of hepatocellular and colitis-associated colon carcinoma, which is associated with a decrease in the proliferation and progression of tumor cells [339]. HIF2α inhibition causes tumor regression in mouse models of primary and metastatic clear cell renal cell carcinoma [340].

Chronic inflammation can lead to conditions that contribute to genomic damage, as well as the emergence and progression of tumors. It is important to note that the production of free radicals, such as reactive oxygen species (hydroxyl radical (OH•) and superoxide (O_2_^−^•)) as well as nitrogen (nitric oxide (NO•) and peroxynitrite (ONOO^−^)), is one of the effector mechanisms, in which the body fights an infection. In addition, it is regulated precisely by inflammatory signaling pathways [341] (elimination of the pathogen by macrophages that primarily occur via the ROS and RNS formation through the plasma membrane-bound nicotinamide adenine dinucleotide phosphate, a reduced form of (NADPH) oxidase [342]). Although radicals are part of the arsenal for fighting infection and are formed to destroy pathogens, it is not surprising that the prolonged exposure to various tissues of highly reactive forms of nitrogen and oxygen, which are released by inflammatory cells, also lead to epigenetic impairments, inhibition of DNA repair mechanisms by cells, accumulation of DNA mutations (such as point mutations, gene deletions or gene rearrangement [321]), and ultimately contributes to some uncontrolled hyperproliferation of transformed cancer cells [322,323]. Therefore, in the blood plasma of patients with cholangiocarcinoma, higher levels of isoprostanes and malonic aldehyde were found, which are markers of DNA oxidation, proteins, and lipid peroxidation [343]. Moreover, it is known that chronic inflammation causes oxidative stress and reduces the antioxidant capacity of cells, not only due to the excessive production of free radicals, but also to the depletion of its own antioxidant defense system. In addition, an increase in the levels of reduced glutathione, superoxide dismutase, and catalase was considered as an effective inhibitor of inflammation in damaged brain cells [344].

ROS and RNS regulate inflammation by activating pro-inflammatory cytokines and NLRP3 inflammasome [345]. In particular, it was found that mitochondrial ROS act as signaling molecules that trigger proinflammatory cytokine production, in particular, IL-1, IL-6, and TNFα [346], which activate NF-κB and STAT3 [347,348,349], the role which was previously mentioned. TNFα has been found to induce tumorigenesis through ROS generation and subsequent DNA damage [350]. Interestingly, ROS also play an important role in the regulation of a family of NOD-like receptors, the NLRP3 inflammasome, which are responsible for the activation of the inflammatory response [351]. The NLRP3 inflammasome has been identified as an oncogene in genomic analyses of non-small cell lung cancer, and in breast [352], head, and neck [353,354] cancers. In addition, melanoma [355] NLRP3 contributes to the progression of malignant neoplasms in all stages, including tumor growth, proliferation, invasion, and metastasis, that also indicate the pathogenic role of inflammasomes in oncogenesis. Interestingly, in NLRP3 deficiency conditions, there is a significant decrease in the IL-18 level in the intestine [356] with a pronounced antitumor effect in colorectal cancer [357]. In addition, mice with NLRP3 knockout show an increase in colorectal cancer metastases in the liver [358]. In human hepatocellular carcinoma tissue, a decrease in the regulation of NLRP3 inflammation was found [359]. In epidermoid carcinoma cells, inhibition of NLRP3 inflammation led to cell death [354].

Therefore, if we summarize the analysis of data on the role of oxidative stress and inflammation in the cancer development, we can say that these are complex, multilevel, closely interrelated processes that can generate each other, forming a vicious closed circle of pathologies. For instance, the main substances linking inflammation with cancer through oxidative stress are prostaglandins and cytokines, which can in turn affect the occurrence of an imbalance between the activity of pro-oxidant and antioxidant enzymes (lipoxygenase, cyclooxygenase, and phospholipidhydroperoxide glutathione peroxidase), that lead to the hyperproduction and accumulation of harmful free radicals, such as hydroperoxides, lipoperoxides, and peroxynitrite. On the other hand, ROS and other overabundant radical contribute to the activation of various transcription factors and hyperproduction of these informational pro-inflammatory molecules, such as cytokines. These processes can all shift the normal metabolism of healthy cells towards the formation of a tumor-like state, which leads to a stop of differentiation and apoptosis avoidance. Therefore, when developing modern effective drugs of multitargeted antitumor action, information on important links in the cancer pathogenesis—oxidative stress and inflammation, should be considered.

## 4. Natural Compounds and Important Targets Associated with Chronic Inflammation and Oxidative Stress in Cancer Treatment

In this section of the review, we tried to combine the data available to date, from experimental works and clinical studies on the use of substances of natural origin, that can modulate the redox balance of the cell and the process of inflammation in cancer therapy.

Researchers have repeatedly attempted to use antioxidants to prevent and treat cancer [360]. Antioxidants can be categorized as natural (dietary), endogenous (glutathione), and synthetic [361]. A large number of reviews exist on the pre- and clinical use of antioxidants and the key mechanisms associated with them [362,363]. A very interesting and intensively developing area of research is the study of natural antioxidants, in which an increasing number of experimental works and reviews are devoted [364,365].

**Figure 9:**
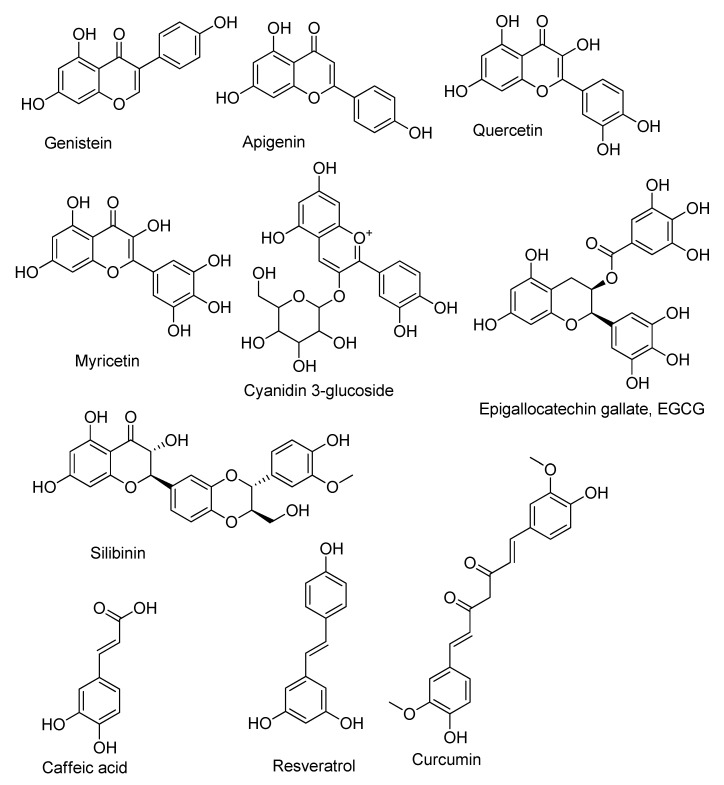
Main investigated phenols and polyphenols.

Natural compounds are secondary metabolites of plants or other living organisms, formed externally for protective purposes. Certainly, they are widely represented in plants that humans eat [366,367,368]. In addition, they belong to the most diverse classes of compounds: Alkaloids, terpenoids, flavonoids, sesquiterpene lactones, steroid compounds, etc. The most extensive and well-known group with antioxidant activities are phenols and polyphenols [369] (Figure 9).

Natural compounds and antioxidants are no exception, and are superior to synthetic compounds with their exceptionally broad activity [370,371]. A large number of these compounds are at various stages of clinical and preclinical research [365,372]. The most studied and interesting flavonoids (Figure 9) are shown below, many of which are at different stages of pre- and clinical studies, the activity which is aimed at redox-sensitive pathways and transcription factors that lead to the overproduction of free radicals, oxidative stress, factors of chronic inflammation, activation or inhibition of transcription factors Nrf2 and NF-κB [373,374,375,376].

Apigenin (4′,5,7-trihydroxyflavone) (Figure 9) is one of the most abundant flavonoid in plants and belongs to the flavone subclass. It is considered safe even at high doses, and its toxicity has not been reported in the literature [377]. Plants belonging to the *Asteraceae* family, such as the genera *Artemisia* [378,379], *Achillea* [380], *Matricaria* [378], and *Tanacetum* [381], are the main sources of this compound.

To date, there is enough in vitro and in vivo experimental data confirming the presence of the therapeutic potential of apigenin in the cancer treatment. Among the mechanisms of antitumor action of apigenin, the ability of this flavonoid to have anti-inflammatory [382] and antioxidant [383] effects, as well as to cause cell cycle arrest at various proliferation stages [384,385] and apoptotic death of neoplastic cells by modulating the expression of apoptotic proteins and signaling pathways [386] are distinguished. The apigenin feature to interfere in the process of carcinogenesis has been shown for a large number of malignant neoplasms [387]. For instance, in [388], it was found that apigenin is able to effectively reduce the expression of the casein kinase 2 in stem cells derived from the cell line of cervical cancer HeLa, an aberrant activation which is detected in various types of cancer [389]. In addition, Zheng et al. demonstrated the ability of apigenin to stop the HeLa cell cycle in the G1 phase, which also correlated with the induction of expression of apoptotic proteins and a decrease in the level of antiapoptotic factors that leads to a significant decrease in the cell survival [390]. In breast cells MCF-10A and MCF-7, apigenin reduced the expression of the enzyme cyclooxygenase 2, which catalyzes the conversion of arachidonic acid into prostanoids and is associated with tumor transformation. Moreover, the sensitivity of pre-transformed cells MCF-10A to apigenin is significantly higher, in order for it to be more effective as a chemopreventive drug rather than a therapeutic [391,392]. Apigenin has also been shown to modulate various anti-inflammatory pathways, in particular, in [393], apigenin weakened the growth of human melanoma cells A375SM, triggering apoptotic cell death by regulating the signaling pathways Akt and MAPK. The treatment of choriocarcinoma cells JAR and JEG3 with this flavonoid inhibited the progression and metastasis of the cells through regulation of the PI3K/Akt and ERK1/2 MAPK signaling pathways. Zhou et al. have also shown that apigenin has antiproliferative, anti-migration, and anti-invasive effects on human lung carcinoma cells A549 by targeting the PI3K/Akt signaling pathway [394]. Recently, in [395], it was found that apigenin may be an appropriate candidate for the treatment of multiple myeloma due to its inhibition of the STAT1/COX-2/iNOS signaling pathway, which is an important mechanism not only for suppressing inflammation, but also for the apoptosis induction. It was reported that by reducing the level of proinflammatory cytokines, apigenin suppressed inflammation and inflammation-induced carcinogenesis in colon cells by regulating the activity of NF-κB and STAT3 [396], and also greatly reduced TNFa levels in the MDA-MB-231 TNBC cell line [397].

Interestingly, apigenin can restore the reduced Nrf2 activity in JB6 P^+^ epidermal cells by demethylation of CpG. The epigenetic mechanism of action of apigenin is realized through a decrease in Nfe2l2 hypermethylation. Induction of miR-101 expression was directed to Nfe2l2 mRNA, while in DNMT1, DNMT3a, and DNMT3b, HDACs are inhibited. Therefore, apigenin may play an important role in cancer prevention and treatment, also through epigenetic modifications [398,399].

Quercetin, 2-(3,4-dihydroxyphenyl)-3,5,7-trihydroxy-4H-chromen-4-one (Figure 9), is a member of the flavonol family. Its compounds are characterized by a 3-hydroxyflavone skeleton. The name has been used since 1857 and comes from the word *quercetum* (oak forest), after *Quercus*. It is easily isolated from a variety of food sources, such as cherries, apples, red wine, etc. Quercetin has a wide range of biological effects: Anti-inflammatory [400,401], anti-infective [402], anticancer/chemopreventive [403,404], neuroprotective [405], hypotensive [406], and blood glucose-lowering properties. Quercetin suppresses the lipopolysaccharide (LPS)-induced production of tumor necrosis factor α in macrophages [407]. Moreover, Bureau et al. showed that quercetin in glial cells could inhibit LPS-induced levels of mRNA, TNFα, and IL-1α. This effect of quercetin led to a decrease in apoptotic death of neuronal cells, which is caused by the activation of microglia [408]. Quercetin suppresses the production of many inflammatory enzymes, such as COX and LOX [409]. It was shown that this flavonoid reduces the production of inflammatory factors in colon cancer Caco-2 cells, which simultaneously correlated with the suppression of the migration and invasive ability of this cell line through TLR4- and/or NF-ĸB-mediated signaling pathway by inhibiting the expression of metastasis-related proteins [410]. A number of studies have found that the treatment of breast cancer cell lines with quercetin triggers the process of tumor cell death along the apoptosis pathway, in particular, by stopping the G1 phase of the cell cycle and suppressing the expression of proliferative activity proteins CyclinD1, p21, Twist, and phospho p38MAPK [411], as well as by inhibiting STAT3 signaling [412]. Blocking of the STAT3 pathway by quercetin also plays an important role in the treatment of glioblastoma, which leads to an effective decrease in the proliferative and migration properties of T98G and U87 cells. Michaud-Levesque et al. have shown that in glioblastoma cells, quercetin leads to a decrease in STAT3 activation and suppression of the expression of the regulated target genes [413]. In a recent study [414], quercetin also reduced IL-6 release and STAT3 phosphorylation in two glioblastoma cell lines U87MG and U373MG, which correlated with a significant decrease in their viability. In [415], quercetin reduced the survival of gastric cancer stem cells by inducing mitochondrial-dependent cell apoptosis through the blockade of the PI3K/Akt signaling pathway and led to a decrease in the mitochondria transmembrane potential, activation of caspase-3 and -9, and suppression of the activity of the apoptosis suppressor Bcl-2, as well as increased Bax and cytochrome C regulation. The antitumor potential of quercetin in prostate cancer therapy is associated with the ability of the flavonoid to exert its anticancer effect by modulating ROS, Akt, and NF-κB pathways [416].

Anti-inflammatory effects were also found for quercetin due to the ability to suppress the expression of matrix metalloprotease-9 (MMP-9) and intercellular adhesion molecule-1 (ICAM-1), as well as block the activation of MAPK and NF-ĸB signaling pathways [417]. Additionally, in [418], a comparison of the anti-inflammatory properties of polyphenols on the NiCl_2_-induced model of migration and invasion in H1975 and A549 human lung cancer cells, the best overall therapeutic efficacy was revealed for quercetin, which can significantly reduce the secretion of cytokines IL-1β, IL-6, TNFα, and IL-10 and the MMP-9 expression. In addition, it suppresses the mRNA and protein expression of TLR4 and Myd88 and NF-ĸB phosphorylation. Interestingly, in an in vivo study on rats with doxorubicin-induced cardiomyopathy, quercetin reduced the biochemical and histological abnormalities caused by the negative effect of cytostatic by increasing the Nrf2 expression [419]. Therefore, the wide range of therapeutic possibilities of quercetin provided by various mechanisms against various cancer types indicates the prospects of considering this flavonoid as an effective tool for the treatment of oncopathologies.

Myricetin (Figure 9) (3,5,7-trihydroxy-2-(3,4,5-trihydroxyphenyl)-4H-chromen-4-one) was first isolated from the bark of tree *Myrica rubra* (Lour.) S. et Zucc and received its name after this plant. Myricetin is one of the most abundant flavonoids in plants as well as plant-derived food products, such as honey, wine, tea, etc. [420,421]. In addition to the previously discussed flavonoids, apigenin and quercetin, myricetin is effective against many diseases, including those associated with oxidative stress, inflammatory and oncological diseases. Its therapeutic effects are related to its influence on many key stages of diseases and signaling pathways that determine the development of these diseases. Here, we only consider some of the mechanisms of action of myricetin on inflammatory and oncological diseases, as well as the main signaling pathways of these diseases on which it exerts its effect. In many LPS-induced diseases, myricetin acts on inflammation by suppressing several key inflammatory factors, primarily TNFα, IL-6, and IL-1α. In this process, two main signaling pathways are involved, in which their endpoints have a nuclear factor NF-κB: AKT-IKK-NF-κB pathway and the TLR4-MyD88-NF-κB signaling pathway [422,423]. In inflammatory diseases, myricetin was found to inhibit IκB-NFκB, Akt, and mTOR signaling pathways, which leads to the decrease in the upregulation of COX-2 and thus decreased levels of many pro-inflammatory cytokines and chemokines [424]. Myricetin can reduce oxidative stress both through its own strong antioxidant action and by influencing the endogenic antioxidant defense system, increasing the activity of antioxidant enzymes, primarily SOD [425].

This was also confirmed in the study by Hassan et al., where myricetin blocks the formation of pro-inflammatory factors, such as Nrf-2, TNFα, NF-κB, etc. [426]. A study by Cho et al. also showed that myricetin regulates the levels of Nrf2-mediated heme oxygenase-1 (HO-1) [427].

Numerous studies have confirmed that myricetin has potent anticancer effects against various types of cancer through a variety of mechanisms that are largely similar to anti-inflammatory mechanisms. Therefore, myricetin acts on the MAPK signaling pathway, regulating the phosphorylation of ERK1/2, JNK, and p38, and inhibits the phosphorylation of Akt [420]. In the study by Li et al., myricetin as well as the previously discussed flavonoids, were shown to have the ability to induce apoptosis in a number of tumor cell lines by decreasing the expression of key signaling pathways, such as glycogen synthase kinase-3 (GSK-3) and Wnt-β-catenin pathways [428]. In the study by Zhu et al., the ability of myricetin was shown to induce apoptosis by modulating the PI3K-Akt-mTOR signaling pathway in glioblastoma U-87 cells [429].

Cyanidin 3-glucoside. Anthocyanins are natural plant dyes and are found in fruits and vegetables in the human diet [430]. Anthocyanins are known antioxidants and are positively charged with respect to the oxygen atom of the C-ring of the main structure of flavonoids (Figure 9) [431], which distinguishes them from other groups of flavonoids. In the last few years, increased attention has been paid to the study of cyanidin 3-glucoside as antioxidant mediators of the Nrf2 pathway. Several studies have demonstrated the antioxidant potential of cyanidin 3-glucoside and its correlation with a decrease in ROS levels, especially in vitro. The two major pro-inflammatory pathways of cytokine production, the MAPK pathway and the NF-κB pathway, were inhibited in cells treated with cyanidin 3-glucoside [432]. The expression of Nrf2-mediated enzymes heme oxygenase-1 (HO-1) and NAD(P)H quinone dehydrogenase 1 (NQO1) increased upon exposure to cyanidin 3-glucoside. Moreover, the treatment with cyanidin 3-glucoside leads to the increased accumulation of Nrf2 in the nucleus, but not in the cytoplasm. This is due to the accumulation under the influence of cyanidin 3-glucoside Nrf2, which is not associated with the trimer complex with Keap1 [433]. Therefore, under the influence of cyanidin 3-glucoside, we received protection against a variety of conditions caused by oxidative stress. The bioavailability and solubility of cyanidin 3-glucoside can be further improved through targeted delivery and nanocrystal or encapsulation technologies [432], which will undoubtedly lead to its more successful application.

Epigallocatechin gallate (EGCG) is composed of ((2R,3R)-5,7-dihydroxy-2-(3,4,5-trihydroxy-phenyl)-chroman-3-yl-3,4,5-trihydroxy-benzoate) [434,435]. Its main disadvantage is its low stability in aqueous solutions and poor solubility in non-polar solvents, which requires special attention and the development of delivery systems, including nanotechnology complexes [436,437,438]. EGCG, as well as other food flavonoids, is well known for its broad spectrum of biological activity and is considered as an anti-inflammatory and antitumor promising substance due to its antioxidant potential [434]. In the study by Sharifi-Rad et al., EGCG is able to modulate the expression of ERK, MAPK, and NF-κB signaling factors [434], as well as increase the level of proapoptotic proteins Bax and Bcl-2, resulting in the downregulated EGFR-RAS-RAF-MEK-ERK signaling pathway, one of the key signaling pathways in the tumor and in apoptosis [439]. The treatment of various tumor cells by EGCG was associated also with the regulation of autophagy. As a result of its action, the cell cycle was stopped through the influence on the balance of the activation/suppression of signaling pathways, in which proteins p1/p21, Kip1/p27, p16/INK4A, cyclin D1, cyclin E, CDK2, and CDK4 were involved [440,441]. The authors of [442,443]] have shown that EGCG is able to activate several caspases and induce apoptosis through the modulation of nuclear factor NF-κB. EGCG in A549 lung adenocarcinoma cells, by modulating reactive oxygen species (ROS), triggers Nrf2/Keap1 signaling and apoptosis [443]. EGCG has the ability to regulate multiple pathways, including Nrf2 activation and NF-κB inhibition [444]. The epigenetic regulation of tumor signaling pathways is currently considered as a promising way to create antitumor drugs [445]. In several studies, the epigenetic activity of EGCG has been demonstrated. At the molecular level, it modulates the expression of DNA methyltransferases (DNMT) and histone deacetylases (HDACs). Similar to the other flavonoids, EGCG has an influence on Nrf2, preventing oxidative damage and inflammation. However, it does not help if Nrf2 is able to form the trimer complex with KEAP1 [446,447].

Resveratrol (5-[E-2-(4-hydroxy-phenyl)ethenyl]benzene-1,3-diol), here referred to as stilbene (Figure 9), is primarily known due to the French paradox, when a relatively low incidence of cardiovascular and oncological diseases associated with the use of red wine was found to be high in resveratrol [448,449]. This natural stilbene has a lot of biological activities: Hepatoprotective [450], antidiabetic [451,452], anticancer [453,454], antioxidant [455], anti-inflammatory [456] action, etc. These unusually broad therapeutic effects are mainly related to its strong antioxidant activity. Therefore, Kabel et al. [457] investigated the effect of resveratrol on an experimental model of kidney cancer in rats. Resveratrol has been shown to have anti-inflammatory and antioxidant activities due to its modulating effect on the Nrf2/HO-1 and STAT3/NF-κB signaling pathway. Clear cell renal cell carcinoma normally shows reduced levels of Nrf2/HO-1. The treatment with resveratrol restored the content of Nrf2/HO-1, which can in turn improve the treatment of this disorder. Of note, resveratrol managed to avoid the metastatic effect. It reduced the expression of xanthine oxidase, IL-6, TNFα, TGF-β1, and LDH in renal tissue. In addition to the chemopreventive and antitumor effects, resveratrol increased the sensitivity of tumor cells to chemotherapeutic drugs. Li et al. [458] found that resveratrol improved the resistance to anthracycline antibiotics by modulating the PI3K/Akt/Nrf2 signaling pathway in promyelocytic leukemia cells (HL-60). Whereas Cheng et al. [459] showed that resveratrol improved the response of pancreatic cancer cells to gemcitabine, increasing the efficacy of gemcitabine in pancreatic cancer therapy, which was associated with the ability of resveratrol to inhibit the expression of nutrient-deprivation autophagy factor-1 (NAF-1) through stimulation signal transmission Nrf2. Moreover, resveratrol dissociates trimer Nrf2-Keap1 and increases Nrf2 translocation into the nucleus [460].

Curcumin, 1E-6E-1,7-bis-(4-hydroxy-3-methoxy-phenyl)-1,6-heptadiene-3,5-dione, was first isolated in 1815 by two German scientists, Vogel and Pelletier, from the well-known spice turmeric (*Curcuma longa* L.) [461]. Turmeric has been used in India for thousands of years as a spice and ayurvedic drug. Curcumin has a wide range of biological activities, including antioxidant, anti-inflammatory, anti-tumor, antiviral, antibacterial, and antidiabetic properties [461,462]. Curcumin provides the selective modulation of multiple cellular signaling pathways associated with various chronic diseases, which strongly suggests that it is an effective multitarget polyphenol [461]. Curcumin modulates several transcription factors, such as Nrf2, β-catenin, NF-κB, inflammatory mediators, and MAPK kinase. Accordingly, it is able to inhibit the JNK-MAPK and ERK-CREB signaling pathways [463]. The mechanism in which curcumin exerts its varied effects is also related to the epigenetic regulation [464]. Several recent studies show that curcumin can modulate some of the epigenetic regulators, histone acetyltransferases (HATs) and histone deacetylases (HDACs) [465,466,467]. This regulation includes not only the acetylation of lysine residues in Nrf2, but the interaction with acetyl “reading” proteins of the bromodomain and extraterminal domain (BET) [398]. The antitumor effect of curcumin is also achieved by the inhibition of many proinflammatory cytokines, such as TNFα and different interleukins [461,468].

Using natural compounds as an example, it has been convincingly shown that targeting redox-sensitive pathways and transcription factors, which leads to the overproduction of free radicals and, as a consequence, cellular oxidative stress, as well as chronic inflammation, opens up great prospects for the prevention and treatment of cancer. Moreover, natural compounds, as substances with an exceptionally wide spectrum of action and that influence many fundamental signaling pathways for the development of inflammation and cancers, represent a promising group for the development of anticancer drugs. However, it is clear that additional experimental research and pharmaceutical development are needed for natural compounds to successfully reach clinical trials.

## 5. Conclusions

Modern data confirm the key role of oxidative stress and chronic inflammation in various stages of tumor development: from initiation to metastasis and the formation of therapeutic resistance. The cancer formation is a multi-step process that includes the mutation and modification of cell growth. The overproduction of highly reactive free radicals and disruption of the cell inherent antioxidant defense system leads to persistent oxidative damage to various macromolecules. This can in turn induce genetic mutations and affect the gene expression, which is important in cancer by modifying the activity of transcription factors. The information presented in this review concerns the following specific mechanisms: (1) Regulation of the inflammation that occurs in various types of cancer by representatives of the antioxidant defense system, (2) the biomarkers of inflammation and oxidative stress recorded in the biological fluids of patients with malignant neoplasms, (3) the use of natural compounds as substances with an extremely broad spectrum of action that influence many fundamental signaling pathways for the development of inflammation and cancer diseases. This can be useful in the development of potential multitarget effective anticancer agents aimed at modulating the cell redox balance and inflammation process and at monitoring the progression of cancer diseases.

## Figures and Tables

**Table 1:** Possible biomarkers associated with oxidative stress and the development of cancer pathology.

Gene	Enzyme	Characteristics of the Enzyme	Ref.
*COX-2*	cyclooxygenase 2	COX-2 is a key enzyme in the biosynthesis of prostaglandins (mainly PGE2) and thromboxanes due to the conversion of arachidonic acid. In malignant neoplasms, COX-2 is an important regulator of angiogenesis, inflammation, and tumor formation, and plays an important role in metastasis, as evidenced by the high level of this enzyme in carcinogenesis.	[73,74,75,76,77,78,79,80]
*NOX-4*	nicotinamide adenine dinucleotide phosphate oxidase subunit 4	Nicotinamide adenine dinucleotide phosphate oxidase subunit 4 (NOX4) is an enzyme expressed by thyroid cells and regulates the production of reactive oxygen species (H_2_O_2_). Aberrant *NOX4* expression contributes to a high rate of DNA mutagenesis and correlates with poor tumor prognosis and low patient survival.	[81,82]
*iNOS*	inducible nitric oxide synthase	Induced nitric oxide synthase is an enzyme responsible for the production of nitric oxide, which is absent in most cells under normal conditions. Aberrant induction of iNOS expression and activation accompanies all stages of carcinogenesis and is also associated with the development of drug resistance phenomenon, a high risk of relapse and death of patients.	[83,84,85]
*CAT*	catalase	Catalase is a key enzyme in the H_2_O_2_ metabolism and active nitrogen forms. Impairment of the expression and localization of this enzyme is characterized with tumor cells in numerous cancer types.	[86,87,88]
*GPx3*	glutathione peroxidase 3	Glutathione peroxidase 3 is a member of the selenoprotein family of glutathione peroxidase and participates in cell protection from oxidative damage ensuring the reduction of organic hydroperoxides and hydrogen peroxide via glutathione. Reduced levels of this enzyme expression are found in tumor samples obtained from patients with various types of malignant neoplasms, which indicate its function as a tumor suppressor.	[89,90,91]
*SOD1*	superoxide dismutase	Superoxide dismutases are a class of enzymes that catalyze the conversion of superoxide radicals into oxygen and hydrogen peroxide. Copper SOD (Cu/ZnSOD, SOD1) is responsible for the regulation of superoxide levels in the intermembrane space of mitochondria, cytosol, and peroxisome, while manganese SOD (MnSOD, SOD2) is the main antioxidant enzyme that absorbs the superoxide radical anion in mitochondria. The enzymatic activity of superoxide dismutases is often reduced in the early cancer stages, while tumor cells contain low levels of SOD proteins.	[92,93,94,95]
*PON*	serum paraoxonase/ arylesterase	The family of antioxidant enzymes paraoxonases consists of three representatives: PON1, PON2, and PON3. The changes in PON status, covering the genotype, activity, and/or expression, were found in cancer patients, as well as in various tumor cell lines. The role of these enzymes in the survival of transformed cells and the formation of chemotherapeutic resistance is shown.	[96,97,98]
